# The BCL-2 arbiters of apoptosis and their growing role as cancer targets

**DOI:** 10.1038/cdd.2017.161

**Published:** 2017-11-03

**Authors:** Jerry M Adams, Suzanne Cory

**Affiliations:** 1 The Walter and Eliza Hall Institute of Medical Research, Parkville, VIC 3052, Australia; 2 Department of Medical Biology, University of Melbourne, Parkville, VIC 3052, Australia

## Abstract

Impaired apoptosis plays a central role in cancer development and limits the efficacy of conventional cytotoxic therapies. Deepening understanding of how opposing factions of the BCL-2 protein family switch on apoptosis and of their structures has driven development of a new class of cancer drugs that targets various pro-survival members by mimicking their natural inhibitors, the BH3-only proteins. These ‘BH3 mimetic’ drugs seem destined to become powerful new weapons in the arsenal against cancer. Successful clinical trials of venetoclax/ABT-199, a specific inhibitor of BCL-2, have led to its approval for a refractory form of chronic lymphocytic leukaemia and to scores of on-going trials for other malignancies. Furthermore, encouraging preclinical studies of BH3 mimetics that target other BCL-2 pro-survival members, particularly MCL-1, offer promise for cancers resistant to venetoclax. This review sketches the impact of the BCL-2 family on cancer development and therapy, describes how interactions of family members trigger apoptosis and discusses the potential of BH3 mimetic drugs to advance cancer therapy.


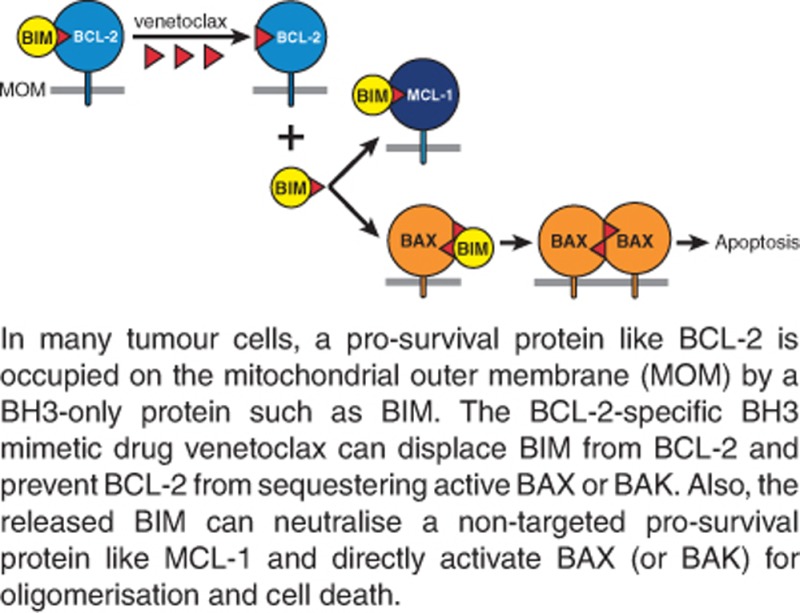


Graphical Abstract

## Facts

- Three factions of the BCL-2 protein family interact to adjudicate whether cells undergo apoptosis. The process is initiated when BH3-only proteins, upregulated by diverse stress signals, engage the surface groove of pro-survival relatives (for example, BCL-2, BCL-X_L_, MCL-1), preventing their constraint of BAX and BAK, which then form oligomers that perforate the outer mitochondrial membrane to elicit caspase activation.
- Diverse tumours have defects in activation of apoptosis because of overexpression of BCL-2 pro-survival proteins or impaired upregulation of BH3-only proteins due to, for example, inactivation of the p53 pathway.
- As a new approach to cancer therapy, drugs termed ‘BH3 mimetics’ that tightly bind the surface groove of certain pro-survival BCL-2 proteins have been developed.
- Venetoclax, a potent BCL-2-specific BH3 mimetic, has been approved for treatment of a refractory form of chronic lymphocytic leukaemia and is under trial for many other malignancies, both as a single agent and in combination with diverse known anticancer agents.
- Genetic data and preclinical studies predict that recently developed BH3 mimetics specifically targeting MCL-1 will be efficacious against multiple haemopoietic malignancies and sensitise some solid tumours to other agents.

## Open questions

- As certain normal cell populations are sensitive to diminished levels of BCL-X_L_ or MCL-1, can an acceptable therapeutic window be found for their inhibitors?
- Given that most current trials of BH3 mimetics have focussed on haemopoietic malignancies, will the new drugs also have a major role in treating solid tumours?
- Which combinations of BH3 mimetics, either with each other or with other targeted or conventional agents, will be most efficacious for different malignancies?
- Can BH3 mimetic therapy provide protracted remissions without the need for long-term treatment?
- Will increased understanding of BAX and BAK oligomers and the elusive apoptotic pore suggest additional ways to target the apoptotic switch for cancer therapy?

The FDA approval in 2016 of venetoclax (also known as ABT-199) for treating a refractory form of chronic lymphocytic leukaemia (CLL) was a significant milestone for cancer research and therapy. The remarkable clinical performance of this drug, designed to mimic natural triggers of apoptosis, capped three decades of research on the BCL-2 protein family. In this review, we reflect on the discovery of BCL-2 and its relatives, summarise how they regulate apoptosis and describe how this knowledge drove the development of BH3 mimetic anticancer drugs. We then sketch the clinical findings that led to FDA approval of venetoclax and discuss its potential and that of other emerging BH3 mimetics, particularly those targeting MCL-1. In addition to the articles in this series,^1, 2, 3, 4, 5, 6^ other recent reviews assess the clinical impact of BH3 mimetics and BCL-2 family function.^7, 8, 9, 10^

## Apoptosis and its first known inhibitor: BCL-2

In vertebrates, apoptosis both shapes the embryo and ensures homeostasis within adult tissues. During apoptosis, cells shrink, fragment their DNA, bleb and break up into ‘apoptotic bodies’ for engulfment by phagocytes.^11^ Importantly, because the plasma membrane is not breached, no inflammation ensues. Apoptosis culminates in activation of cysteine proteases called caspases that cleave vital cellular proteins. Caspases are activated through either the ‘*extrinsic*’ apoptosis pathway, triggered by engagement of cell surface ‘death receptors’ of the tumour necrosis factor (TNF) receptor family, or the ‘*intrinsic*’ pathway, initiated by diverse cellular stresses. The BCL-2 protein family regulates the latter, by controlling the integrity of the mitochondrial outer membrane (MOM).

BCL-2 was the first inhibitor of apoptosis to be discovered, in any species. The gene was found linked to the immunoglobulin heavy chain gene locus by the t(14;18) chromosome translocation that hallmarks human follicular lymphoma (FL).^1^ Although BCL-2 seemed likely to be a new oncoprotein, its sequence provided no clues about its function. In 1988, Vaux *et al.*^12^ solved the mystery by showing that lymphocytes forced to express elevated BCL-2 resisted apoptosis when deprived of their requisite cytokine. This seminal study revealing BCL-2 as the prototypic inhibitor of cell death also established, for the first time, that cytokines signal cell survival and proliferation by different pathways and that impaired apoptosis contributes to malignant transformation.

*BCL-2* transgenic mice reinforced and extended these observations. The excess lymphocytes they accumulated had failed to die in response to physiological cues and resisted diverse cytotoxic agents, including chemotherapeutic drugs.^13, 14, 15, 16^ Notably, mice co-expressing *BCL-2* and *myc* transgenes developed lymphomas markedly faster than littermates expressing either transgene alone,^17^ validating *BCL-2* as an oncogene. Clarifying the basis for the synergy with *myc*, enforced MYC expression proved to stimulate apoptosis.^18, 19^ Thus, by blocking apoptosis, BCL-2 removes a critical brake on MYC-driven proliferation and oncogenesis.

As t(14;18) translocations can be detected in B cells of healthy humans,^20^ follicular lymphoma requires mutations additional to *BCL-2* translocation, and perhaps also chronic T-cell stimulation.^21^ Several other human malignancies express elevated BCL-2 because of diverse mechanisms. Notably, the high BCL-2 in CLL reflects loss of microRNAs that normally dampen translation of its messenger RNA.^1^

## The BCL-2 protein family

**Figure 1:**
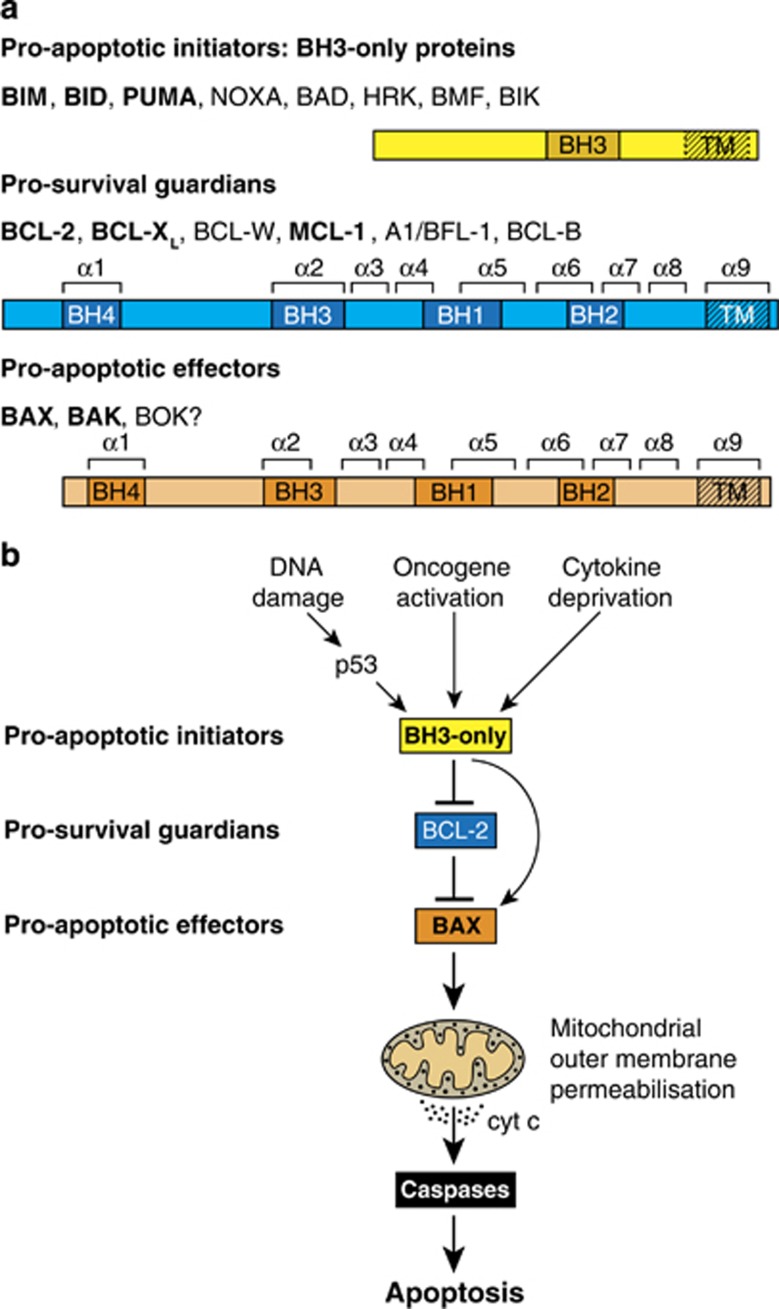
The BCL-2 protein family. (**a**) The initiator, guardian and effector factions of the family. Domains of shared *B*CL-2 *H*omology (BH), and the nine *α*-helices in the multi-BH domain members are indicated. (Effectors BAX and BAK, and the related BOK, have a BH4 domain if both structural and sequence homology are considered.^29^) BOK seems to drive apoptosis only in special circumstances.^6^ Faction members most important for controlling apoptosis are in bold. All multi-BH domain family members and some BH3-only proteins (BIM, BID, BIK, HRK) have a C-terminal transmembrane (TM) domain for anchoring to organelles, most notably the MOM. (**b**) How the BCL-2 protein family controls cell life and death is shown. In healthy cells, the pro-survival guardians prevent activation of BAX and BAK, at least in part by binding the BH3 domain (*α*2) of any destabilised BAX or BAK monomers. Various stress signals activate BH3-only proteins that avidly bind their pro-survival relatives, preventing their constraint of BAX or BAK. In addition, certain BH3-only proteins, namely BIM, cleaved (active) BID and probably PUMA, can directly activate BAX and BAK, which then homo-oligomerise and permeabilise the MOM, releasing cytochrome *c* to initiate caspase activation and cellular demolition. Modified, with permission, from Figure 1 of Cory *et al.*^8^

Vertebrate proteins related to BCL-2 bear from one to four *B*CL-2 *H*omology (BH) domains and fall into three functional factions (Figure 1a). The closest BCL-2 relatives (BCL-X_L_, BCL-W, MCL-1, A1/BFL-1 and, in humans, BCL-B) promote cell survival, but BAX and BAK (and possibly BOK) instead promote cell death, as do distant relatives termed BH3-only proteins, because their only homology to BCL-2 (or each other) is the BH3 domain, through which they engage multi-BH domain relatives.

Interactions between these factions determine whether cells live or die (Figure 1b). In healthy cells, pro-survival proteins prevent apoptosis by sequestering any activated pro-death relatives. However, upon diverse cellular stresses (e.g., cytokine or nutrient deprivation, DNA damage or oncogene activation), BH3-only proteins are upregulated and avidly bind the pro-survival proteins, preventing their constraint of BAX and BAK. In addition, certain BH3-only proteins, particularly BIM and BID and probably PUMA, can directly activate BAX and BAK, prompting their homo-oligomerisation and MOM permeabilisation. Cytochrome *c* then leaks into the cytosol, where it helps form the apoptosome that activates caspase-9. In turn, caspase-9 activates effector caspases 3, 6 and 7 that cleave vital cellular proteins, ensuring cellular demolition.

Curiously, despite the evolutionary conservation of many players, cell death regulation differs significantly between invertebrates and vertebrates.^2^ Thus, the worm *Caenorhabditis elegans* has a BCL-2 homologue (CED-9) and a BH3-only antagonist (EGL-1) but no BAX/BAK homologue, and CED-9 does not act by maintaining mitochondrial integrity. Furthermore, *Drosophila* BCL-2-related genes have only minor roles in fly cell death.

### Critical roles of vertebrate family members

Gene ablation revealed that the widely expressed BAX and BAK are functionally redundant, apoptosis requiring one or the other.^22^ In contrast, individual pro-survival proteins vary in abundance in different cell types, producing differential dependencies.^3, 23^ For example, BCL-2 is crucial for survival of circulating lymphocytes; BCL-X_L_ for neurons, erythroid cells and platelets; and MCL-1 for many haemopoietic (and other) cell types, including stem and progenitor cells. The BH3-only proteins, which are regulated by multiple mechanisms, mediate the responses to various cytotoxic insults.^23, 24^ For example, whereas DNA damage upregulates p53 protein, which induces expression of *PUMA* and *NOXA*, cytokine deprivation relies mainly on BIM and PUMA.^23^ Just as the pro-survival proteins are oncoproteins, several BH3-only proteins can be tumour suppressors.^23, 24, 25^

### Rules of engagement between factions

As first found for BCL-X_L_,^26, 27^ all multi-BH domain family members comprise a globular bundle of nine *α*-helices, and all display a hydrophobic surface groove created largely by the conserved BH1, BH2 and BH3 domains.^10, 28, 29^ In contrast, most BH3-only proteins are intrinsically unstructured until their BH3 domain forms an amphipathic helix upon engagement of a multi-BH domain partner. The exception is BID, which resembles a multi-BH domain protein and requires cleavage to expose its BH3 domain.^29^

**Figure 2:**
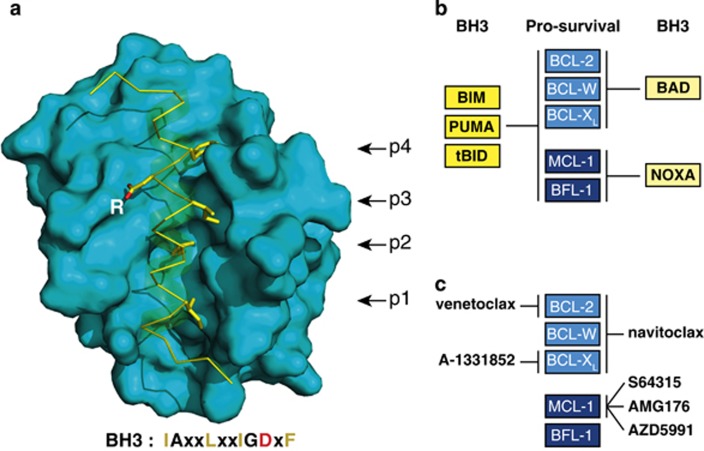
Interaction of BCL-2 family members. (**a**) The canonical BH3/surface groove interaction in the family. Structure of BCL-X_L_ (blue surface representation) bound to the amphipathic helical BH3 peptide of BIM (a yellow ribbon indicates its helical structure)^27^ (PDB/3FDL), with its N terminus at the bottom. Underneath the protein is a consensus BH3 sequence of the pro-apoptotic proteins (x denotes nonconserved residues). The four key hydrophobic amino acids (yellow) of the Bim BH3 peptide that bind to pockets p1 to p4 in BCL-X_L_ are highlighted, as is the invariant aspartic acid (D) (oxygens in red) that binds to a conserved arginine (R) in BCL-X_L_. BIM or BID BH3 peptides associate with the grooves of BAX or BAK through contacts resembling those with their pro-survival relatives (as above) but include additional contacts that contribute to their activator function.^41, 42, 43, 44^ (**b**) Selective association of BH3-only proteins with their pro-survival relatives. Whereas BIM, PUMA and tBID bind promiscuously, BAD and NOXA have restricted targets, as indicated. (**c**) BCL-2 pro-survival targets of current BH3 mimetic drugs. A BH3 mimetic engages the surface groove of the targeted pro-survival protein(s) in a manner akin to their natural antagonists, as in (**a**), but usually involving only pockets p2 and p4. In cells, binding of the compound to their pro-survival target(s) releases any bound BH3-only proteins and prevents the targeted pro-survival protein(s) from restraining BAX and BAK. Note that preclinical studies were reported on Servier MCL-1 inhibitor S63845,^115^ but the clinical candidate from Novartis/Servier is the more advanced derivative S64315. Modified, with permission, from Figure 2 of Cory *et al.*^8^

The canonical interaction between family members is the binding of the BH3 domain of a BH3-only protein, or of BAX or BAK, to the surface groove of a multi-BH domain protein (either pro-survival or pro-apoptotic).^30^ As Figure 2a illustrates for a BIM BH3 peptide bound to BCL-X_L_, four conserved BH3 hydrophobic residues project into hydrophobic groove pockets p1-p4, and a BH3 aspartic acid (D) pairs with an adjacent conserved arginine (R) in the groove.

BH3-only proteins vary in affinity for different pro-survival relatives^31, 32, 33^ because of sequence differences within both the BH3 domain and groove.^29^ Whereas BIM, PUMA and cleaved BID (tBID) avidly bind all five pro-survival relatives, BAD binds only BCL-2, BCL-X_L_ and BCL-W, and NOXA engages only MCL-1 or A1/BFL-1 (Figure 2b). BH3-only proteins that bind all pro-survival proteins (BIM, PUMA, tBID) are more potent killers than those with restricted binding profiles. Importantly, however, NOXA plus BAD kills potently, suggesting that efficient killing requires neutralisation of all pro-survival members in the relevant cell type.^32^

The BH3 domains of activated BAX and BAK also show preferences: BAK is restrained primarily by BCL-X_L_, MCL-1 and A1/BFL-1,^34^ whereas BAX is controlled by all pro-survival members.^35^

The outcome of BH3 domain binding differs dramatically between a pro-survival and a pro-apoptotic multi-BH domain protein. The BH3 complexes with pro-survival proteins are stable, whereas those with BAX or BAK are fleeting and elicit remarkable conformational changes, as detailed below.

## The life/death switch on the MOM

In healthy cells, pro-survival and pro-apoptotic multidomain proteins are distributed between the cytosol and intracellular membranes, with the MOM being the primary site regulating apoptosis. Whereas BCL-2 and BAK are predominantly integral MOM proteins, BAX is mainly cytosolic, with its hydrophobic tail (*α*9) partially sequestered in its groove.^28^ Apoptotic cues lead to release of *α*9, which attracts BAX to the MOM, although it can be ‘retro-transposed’ back to the cytosol by pro-survival proteins like BCL-X_L_.^36^ BAK behaves similarly, but its equilibrium strongly favours MOM insertion.^37^ Both BAX and BAK initially associate with the MOM in a large complex with VDAC2,^38, 39, 40^ a minor isoform of the voltage-dependent anion channel that mediates nutrient and ion transport through the MOM.

**Figure 3:**
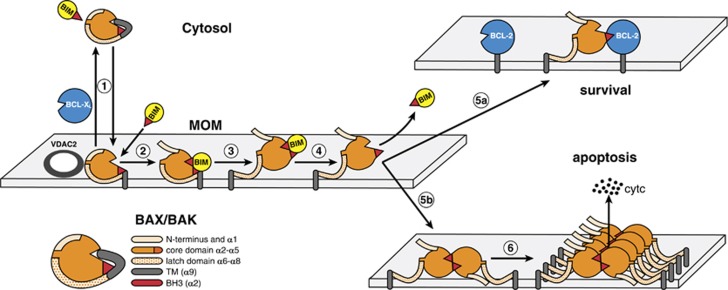
Model for life/death decisions on the MOM, showing how pro-survival family members constrain BAX (and BAK) and how BH3-only proteins (here BIM) drive their activation. In healthy cells, monomeric BAX shuttles between the cytosol and MOM, where VDAC2 acts as a receptor (also for BAK), although pro-survival relatives (here BCL-X_L_) can ‘retro-translocate’ MOM-bound BAX back to the cytosol.^36^ Upon apoptotic signalling, to allow more BAX to move to the MOM (step 1), where most BAK molecules reside, an activator BH3-only protein such as BIM may transiently engage a BAX ‘rear site’ involving helices *α*1 and *α*6,^130, 131^ thereby releasing the C-terminal trans-membrane (TM) domain (*α*9) from its surface groove to enable MOM binding. Then, groove binding by the activator drives release of the N terminus and *α*1 of BAX or BAK (pale orange) (step 2) and all subsequent activation steps for both the MOM-bound effector proteins. The most dramatic change is the unfolding of BAX and BAK that separates their ‘latch’ domain (*α*6–*α*8; speckled orange) from their ‘core’ domain (*α*2–*α*5)^41, 43^ (step 3); this ejects the BH3-only activator (BIM here) and exposes the BH3 domain of BAX or BAK (*α*2, red triangle) (step 4). If pro-survival proteins are available to bind the exposed BAX (or BAK) BH3 domain, apoptosis aborts (step 5a). However, if pro-survival proteins are largely occupied by BH3-only proteins, the unfolded BAK or BAX monomers form homodimers through reciprocal BH3/groove interactions of their core domains (step 5b).^41, 43, 46, 47^ The core dimers are the central unit of the BAX and BAK homo-oligomers,^46, 47, 132^ but how they associate into oligomers (step 6) remains uncertain, as does how the oligomers drive MOM permeabilisation (see text). Modified, with permission, from Figure 3 of Cory *et al.*^8^

The switch from survival to apoptosis is triggered when BH3-only proteins reach concentrations sufficient to both neutralise their pro-survival relatives and activate BAX or BAK, culminating in BAX/BAK homo-oligomerisation and MOM pore formation. Direct activator BH3-only proteins such as BIM and tBID induce multiple conformational changes in BAX and BAK (Figure 3).^41, 42, 43, 44^ As well as dislodging the BAX transmembrane domain (*α*9) from its surface groove,^28^ these include release of the N-terminal segment (including *α*1) of both BAK and BAX.^45^ Then, remarkably, BAX and BAK unfold into an N-terminal ‘core’ (*α*2–*α*5) and a C-terminal ‘latch’ (*α*6–*α*8).^41, 43^ This metamorphosis displaces the activating BH3-only protein and exposes the BH3 domain of BAX/BAK (*α*2), creating a critical decision point (Figure 3). If pro-survival proteins such as BCL-2 are available to bind the BH3 domain of activated BAX/BAK, apoptosis aborts (Figure 3, upper right). However, if the pro-survival proteins are largely occupied by BH3-only proteins, the cores of unfolded BAK or BAX monomers instead form homodimers through reciprocal BH3/groove interactions (Figure 3, lower right).^41, 43, 46, 47^

Though the evidence is now compelling that direct engagement of BAX or BAK by a BH3-only protein can trigger their activation, this interaction may be catalytic rather than obligatory because in a mammalian cell line engineered to lack all the BCL-2 pro-survival proteins and all BH3-only proteins, BAX and BAK spontaneously engaged the MOM, oligomerised and evoked apoptosis.^48^ Thus, restraint by the pro-survival faction is crucial to prevent unwarranted cell death provoked by BAX and BAK.

### The quest for the elusive apoptotic pore

As all mutations impairing BAX or BAK dimerisation impair MOM permeabilisation,^46, 49^ BAX/BAK homodimers and probably also the larger homo-oligomers they form must be required.^4^ However, how are the reciprocal dimers linked into oligomers? Although they can be coupled via *α*6,^50, 51^ *α*3 and *α*5,^52^ *α*9^53, 54^ or *α*1,^55^ all these proposed linkages are weaker than those that maintain BH3 domain-groove dimers, and none as yet appears essential for oligomerisation, prompting a recent proposal that disordered dimer clusters suffice to disrupt the MOM.^55^

Most current apoptotic pore models^4, 52, 55, 56, 57, 58^ suggest that lipids partially wall the pores and that the oligomers do not have a simple structure. Contrary to the long-standing ‘umbrella’ model in which a BAX *α*5–*α*6 hairpin penetrates the MOM,^26, 59^ helices *α*4 and *α*5, which form the hydrophobic ‘bottom’ of the stable BH3/groove dimer,^41, 43^ appear to lie ‘in-plane’ with the MOM and sink only into its outer leaflet, as do the flexible *α*6, *α*7 and *α*8 helices.^55, 56^ Their shallow insertion would expand the outer leaflet relative to the inner leaflet, promoting tension that induces a nascent lipidic pore that the dimers or oligomers may stabilise by sliding over the rim.^56, 58^ Indeed, in the ‘clamp model’, a core dimer sits on the rim with its two flexible *α*6–*α*8 ‘arms’ projecting on the inner and outer leaflets, and the two *α*9 helices penetrate the bilayer from both faces to stabilise the pore.^57^

High-resolution imaging is beginning to reveal the pores. Cryo-EM showed nanogold-labelled activated BAX edging large pores in liposomes.^60^ Super-resolution microscopy on activated BAX in mitochondria also revealed large arcs and rings,^61, 62^ indicative of pores,^62^ and atomic force microscopy of lipid bilayers revealed heat-activated BAX oligomers around huge holes 20–80 nm in diameter.^61^ Lining such rings might require >40 BAX dimers. However, these studies cannot distinguish a fully BAX-lined pore from a partially lipidic rim braced by BAX helices. Irrespective, it seems that BAX oligomers expand limitlessly rather than forming a pore of defined, or even preferential, size.

## Rationale for BH3 mimetic anticancer drugs and their development

Many tumours, especially those refractory to therapy, express elevated levels of one or more pro-survival family member,^7, 63^ and many carry mutations that cripple induction of BH3-only proteins. In particular, most tumours (~90%) have mutations that inactivate the p53 protein, delete its gene or impair upstream regulators, preventing p53 induction of PUMA and NOXA to drive apoptosis.^64^ In addition, 17% of mantle cell lymphoma cell lines have homozygous deletions of *BIM*,^65^ and many Burkitt lymphomas harbour epigenetically silenced *BIM* or *PUMA* alleles.^66, 67^

Although such changes can make cancer cells resistant to cytotoxic agents, including radiation and chemotherapeutics, most tumours retain the core apoptotic machinery, suggesting that organic molecules mimicking BH3-only proteins might switch on apoptosis. However, the long, shallow and primarily hydrophobic groove of the pro-survival proteins made the challenge daunting. Indeed, most of the putative BH3 mimetics initially reported had relatively modest affinity for pro-survival proteins and few passed the definitive test of requiring BAX or BAK to kill cells.^68, 69^

The first *bona fide* BH3 mimetic, ABT-737, was developed by Abbott Laboratories (now AbbVie) in a decade-long *tour de force* using NMR fragment screening, structural biology and medicinal chemistry to enhance affinity and reduce serum binding.^70^ Like the BH3-only protein BAD (Figure 2b), ABT-737 has low nanomolar affinity for BCL-2, BCL-X_L_ and BCL-W but negligible affinity for MCL-1 or A1/BFL-1, as does the orally bioavailable derivative navitoclax (ABT-263) developed for the clinic.^71^ The crystal structures of BCL-X_L_ binding ABT-737^72^ and BCL-2 binding ABT-263^73^ revealed intriguing differences to their complexes with a natural ligand like BIM.^27^ Although two hydrophobic moieties of the compounds engage the p2 and p4 groove hydrophobic pockets (Figure 2a), the p2 pocket is penetrated much more deeply than by the BIM invariant BH3 leucine, revealing unanticipated groove plasticity.

Because BCL-X_L_ controls platelet lifespan,^74^ ABT-737 and navitoclax provoke acute dose-limiting thrombocytopaenia.^75, 76^ Efforts to circumvent this problem led to the development of the BCL-2-specific venetoclax (ABT-199)^73^ that spares platelets.^73, 77^ Venetoclax differs from navitoclax primarily by engaging the p4 pocket in a manner that exploits a difference between BCL-2 (Asp103) and BCL-X_L_ (Glu96).^73^

Other BH3 mimetics are emerging.^9^ They include another BCL-2-specific inhibitor (Servier’s S55746); the BCL-X_L_-specific WEHI-539^78^ and its more potent derivatives A-1155463 and A-1331852;^79^ and, most recently, three MCL-1-specific inhibitors (see below) (Figure 2c).

### Many tumour cells are primed to die

**Figure 4:**
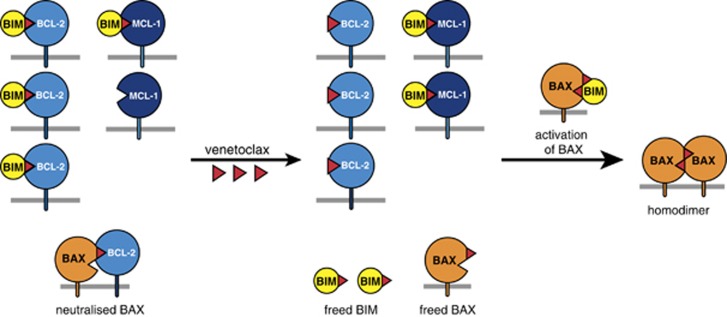
Killing of ‘primed’ cancer cells by BH3 mimetics. Because of the many stresses imposed by the tumourigenic process, such as abridged cell cycle checkpoints, hypoxia or altered metabolism, many cancer cells have been selected for elevated expression of pro-survival proteins, here BCL-2 and MCL-1. These stresses will have elevated the level of BH3-only proteins such as BIM, much of which may be in complex with BCL-2 (top left). Hence, paradoxically, the ‘primed’ cancer cell, despite elevated BCL-2, can be nearer the apoptotic threshold than normal cell counterparts.^80^ (see text and Letai^5^ in this series.) When such cells are exposed to a BCL-2-specific BH3 mimetic like venetoclax (red triangles), BIM is displaced from BCL-2, as is any BAX previously sequestered by BCL-2 (bottom left). The freed BIM can then sequester any unoccupied non-targeted pro-survival protein (here MCL-1) (centre); it can also activate inactive BAX (or BAK) monomers (right), thereby facilitating apoptosis. Thus, the presence in a primed cancer cell of abundant complexes of a BH3-only protein like BIM with a pro-survival partner renders the cell more vulnerable to apoptosis. Once activated BAX monomers build up on the MOM, each can insert its exposed BH3 (*α*2) into the groove (*α*3–*α*5) of another activated monomer, generating a ‘symmetric’ homodimer (right).^41, 43, 46^ These dimers can then oligomerise and permeabilise the MOM, releasing cytochrome *c* (cyt *c*) from the intermembrane space to trigger caspase activation (see Figure 3). Modified, with permission, from Figure 5 of Cory *et al.*^8^

Paradoxically, many tumours with elevated levels of a pro-survival protein are nonetheless sensitive to cytotoxic therapies, including BH3 mimetics. Indeed, they seem ‘primed to die’, that is, more sensitive than their normal counterparts.^5, 80^ The basis of priming appears to be that the mutations and stresses suffered by a cell *en route* to malignancy upregulate BH3-only proteins such as BIM, imposing selective pressure for elevated levels of pro-survival proteins. Consequently, pro-survival proteins loaded with potent BH3-only proteins like BIM put the cells in many tumours on the brink of apoptosis (Figure 4).^81^ This concept explains how a cancer cell with elevated BCL-2 can be *more* susceptible to apoptosis than a normal cell with lower BCL-2.

Notably, in a primed tumour cell, the impact of a BH3 mimetic can extend beyond the targeted protein. Thus, in Figure 4, although venetoclax targets only BCL-2, the BIM it frees from BCL-2 can engage the non-targeted MCL-1 or BAX or BAK, enhancing sensitivity.^5, 81, 82^ Priming, which can be assessed by the sensitivity of mitochondria in permeabilised cells to disruption by BH3 peptides, often correlates with responses to cytotoxic chemotherapy.^83, 84^

Unexpectedly, unlike malignant and neonatal tissues, many normal adult tissues (particularly brain, heart, kidney) are refractory to both BH3 mimetic and conventional cytotoxic therapies because their apoptotic systems are enfeebled, in part by a dearth of BAX and BAK.^85^

### The therapeutic potential of BH3 mimetics

#### Navitoclax trials show promising efficacy

Navitoclax was the first authentic BH3 mimetic to enter clinical trials, after extensive preclinical studies on ABT-737 and navitoclax had established their efficacy and mechanism of action.^68, 70, 71, 86^ Navitoclax proved active in malignancies with high BCL-2, such as CLL and FL.^75^ Although the predicted rapid drop in platelets^74^ precluded determining the maximal response rate, circulating tumour cells were reliably reduced in CLL and 35% of patients had an objective response.^75^ Although responses in FL were very limited, combining navitoclax with the CD20 antibody rituximab proved safe and markedly improved response rates in both CLL and FL.^87^

#### Venetoclax: the first BH3 mimetic to enter routine clinical practice

The accelerated FDA approval of venetoclax followed highly encouraging phase 1^88^ and phase 2^89^ clinical trials indicating that daily oral venetoclax is effective against relapsed and refractory CLL, including disease resistant to DNA-damaging chemotherapy and having poor prognostic features. Indeed, some of these patients had exhausted all other credible treatment options. Of those receiving therapeutically effective levels (150 to 1200 mg/day), 79% had an objective clinical response and 20% a complete remission.^88^ Like navitoclax,^75^ venetoclax evoked canonical apoptosis features in CLL cells, verifying the mechanism of action.^90^ BH3 mimetics act downstream of p53 (Figure 1b) and, as expected, their efficacy proved independent of p53 status.^75, 88, 89, 90^ The high response rate in CLL patients with deletion 17p^89^ considerably exceeded those obtained previously for combinations of monoclonal antibodies and chemotherapy.^88^ Thus, venetoclax significantly advances CLL therapy.^5^

In the first venetoclax trial, the precipitous tumour decimation in several CLL patients with a large tumour burden provoked tumour lysis syndrome,^88^ which arises when tumour destruction exceeds the body’s capacity to remove cell debris. This potentially fatal syndrome is now avoided by ramping up the dosage over a month, from a low level to the target dose.^88^ Other toxicities include mild gastrointestinal side effects and neutropaenia, both of which can be well managed clinically.^88^

Other B lymphomas with high BCL-2 that respond to venetoclax as a single agent include mantle cell lymphoma and, less frequently, follicular lymphoma, myeloma and some diffuse large B-cell lymphomas.^91^ Responses in FL and myeloma often required higher doses than in CLL, probably because MCL-1 and BCL-X_L_ are more abundant than in CLL.^92^ Relapsed or refractory acute myeloid leukaemia (AML) has also shown modest single agent responsiveness (19%).^93^

#### Venetoclax sensitises to other therapies

Preclinical data^73, 79, 94, 95^ predict that rational combination therapies should greatly boost the clinical impact of venetoclax. Accordingly, in CLL and B-cell lymphomas, venetoclax is under trial with anti-CD20 antibodies, with or without genotoxic chemotherapy; in CLL and mantle cell lymphoma, with ibrutinib that inhibits Bruton’s tyrosine kinase; and in multiple myeloma, with proteasome inhibitor bortezomib and steroids (see https://clinicaltrials.gov/ct2/results?term=venetoclax).

The most advanced available data are from venetoclax plus rituximab in relapsed CLL. Exciting results indicate an 86% response rate and complete remissions in 51%, over twice that with venetoclax alone.^96^ Moreover, in 80% of complete responders and 57% of all treated patients, the bone marrow exhibited no minimal residual disease. Notably, all 11 patients without minimal residual disease who stopped treatment remain progression free,^96^ raising the exciting prospect of protracted remissions without continuous venetoclax therapy.

Venetoclax combination therapy should have important applications beyond lymphoid malignancies. Clinical trials in treatment-naive AML with venetoclax plus demethylating agents (decitabine or azacitidine) have yielded highly promising preliminary results, including 38% complete remissions.^97^ Furthermore, in preclinical AML models, targeting both BCL-2 and MCL-1 eradicated disease, and venetoclax synergistically enhanced the activity of cytarabine and idarubicin.^98^ Similarly, AML cells with mutated isocitrate dehydrogenase 1 or 2 are BCL-2 dependent, and preclinical studies showed that this AML subset was sensitive to venetoclax plus agents that disrupt mitochondrial electron transport.^99^

Although inhibitors of the BCR-ABL kinase, for example, nilotinib, have revolutionised treatment of chronic myeloid leukaemia (CML), cures are rare, because the ‘leukaemia stem cells’ remain, and a refractory blast crisis can ensue. In a mouse CML model, however, venetoclax plus nilotinib eliminated the leukaemia stem cells and extended mouse survival.^100^ This drug combination even killed human CML cells in blast crisis.^100^ Thus, the efficacy of some BH3 mimetics may reflect their eradication of ‘cancer stem cells’.

Currently, nearly 50 clinical trials of venetoclax are underway in different tumour settings. Although the focus has been on haemopoietic malignancies, trials for solid tumours are eagerly awaited. Pertinently, combining venetoclax with the anti-oestrogen tamoxifen markedly improved responses of ER^+^ breast cancer xenografts that express high BCL-2.^101^ Of greatest moment would be identifying a therapy that eliminated metastasis, the major killer in cancer.

#### Exciting prospects for targeting MCL-1

Cancer researchers have long sought an effective MCL-1 inhibitor. The elevated MCL-1 found in many tumour types^63^ is implicated in therapy resistance, including of some breast and lung cancers,^102, 103^ and many studies^68, 104, 105^ show that MCL-1 mediates resistance to navitoclax and venetoclax, as their complementary targets suggest (Figures 2b and c). Most compellingly, conditional gene deletion (or knockdown) demonstrates that MCL-1 is required for the sustained growth of AML driven by MLL-fusion genes,^106^ MYC-driven B lymphoma,^107^ TP53^−/−^ thymic lymphoma,^108^ BCR-ABL-driven acute lymphoblastic leukaemia^109^ and myeloma.^110^

Valid concerns about the safety of MCL-1 inhibition have arisen from reports of MCL-1 dependence for mouse cardiomyocytes,^111^ hepatocytes^112^ and neurons.^113^ However, *mcl-1*^+/−^ mice, which should mimic 50% inhibition, are normal and healthy, suggesting that a suitable therapeutic window may well be found. Furthermore, MCL-1 functions that are independent of BH3 binding^114^ may contribute to the knockout results.^3^

Developing a specific MCL-1 inhibitor has been challenging,^9^ but very recently three highly potent (sub-nanomolar) and selective inhibitors have emerged. Preclinical data on the Servier inhibitor S63845 are very impressive.^115^ Notably, 92% of the multiple myeloma cell lines tested were highly or moderately sensitive, as were 83% of human lymphoma lines, all tested AML cell lines and half the primary AML samples. This sensitivity translated into markedly extended mouse survival in xenograft and transgenic models. Furthermore, certain solid tumour cell lines, particularly those with low BCL-X_L_, were sensitised to conventional therapies. For example, S63845 showed synergy with current therapies in preclinical models of two types of breast cancer.^116^ Importantly, mice tolerated S63845 well, with normal tissues unaffected at doses that eradicated MYC-driven mouse lymphomas.^115^ Thus, a therapeutic window seems likely. Preliminary preclinical reports on the other two new MCL-1 inhibitors are also exciting. Amgen AMG 176 was potent in myeloma, AML and non-Hodgkin’s lymphoma cell lines and xenografts, and a phase 1 myeloma trial is underway.^117^ Myeloma was also particularly sensitive to AstraZeneca AZD5991: a single tolerated dose achieved 100% tumour regression in xenografts.^118^ Clinical findings on the tolerability and efficacy of these three new inhibitors will attract close interest.

#### Potential of other emerging BH3 mimetics

Although no trials are underway, a BCL-X_L_-specific BH3 mimetic may well have clinical utility, as many solid tumours, especially colorectal cancer,^119^ have elevated BCL-X_L_ that correlates with chemoresistance in cancer cell lines.^120^ Selective BCL-X_L_ inhibitors (A-1331852, the most potent) were well tolerated in mice and modestly enhanced docetaxel efficacy against diverse solid tumour xenografts.^79^ As mice lacking one *BCL-X*_*L*_ allele are healthy^121^ and ramped dosing can largely control the expected thrombocytopaenia,^87^ a therapeutic window for BCL-X_L_ inhibition seems likely.

No specific inhibitors have yet been developed against BCL-W, BFL-1 or the little studied BCL-B. BCL-W, recently implicated in maintenance of MYC-driven B lymphomas,^122^ should be a safe target, because *BCL-W*^−/−^ mice are normal, apart from male sterility.^123^ BFL-1 (called A1 in mice) should also be a good target, as it contributes to chemoresistance,^105, 124^ and mice lacking all three A1 isotypes are normal and healthy.^125^

#### Potential of directly activating BAX or BAK

In principle, any compound directly activating BAX or BAK could be a lead to an effective anticancer agent, given an acceptable therapeutic window. Proof-of-principle studies suggest that compounds engaging either the BAX ‘rear’ site^126^ or its groove^127^ might have promise, as should other sites driving BAX or BAK activation, for example, the *α*1–*α*2 loop,^128^ or a site at the junction of the *α*3–*α*4 and *α*5–*α*6 hairpins that sensitises BAX activation.^129^

## Concluding remarks

The remarkable success of BH3 mimetics has broken new ground in drug development by demonstrating that protein–protein associations can be targeted with high potency and exquisite specificity, although requiring much larger compounds than enzymes. With highly selective inhibitors now in hand for the three major pro-survival family members, namely BCL-2, BCL-X_L_ and MCL-1, cancer researchers and clinicians can rapidly expose the vulnerabilities of multiple cancer types and explore the efficacy of BH3 mimetics not only as single agents but also in combination with each other or with other targeted and conventional agents.

As most, if not all, conventional cytotoxic agents kill through the apoptotic switch governed by the BCL-2 family, what advantages do BH3 mimetics offer for cancer therapy? First, their direct engagement of the apoptotic machinery is more efficient and selective. Second, because they act downstream of p53 (Figure 1b), even the vast majority of tumours with a defective p53 pathway remain vulnerable.^90^ Third, the oncologist can focus therapy on the pro-survival target(s) to which a particular tumour is ‘addicted’. Fourth, at least with CLL, venetoclax responses appear unusually effective and durable, possibly because it can target tumour-initiating cells, as shown for CML.^100^ Fifth, many tumours seem particularly vulnerable to BH3 mimetics because of their abundant complexes of BH3-only proteins with a pro-survival partner (Figure 4). Sixth, unlike radiotherapy and other DNA-damaging agents, which undoubtedly increase mutational load, BH3 mimetics are not mutagens. Seventh, their mechanism of action is well understood, whereas that of many conventional agents is not. Finally, unlike therapies linked to specific oncogenic mutations or particular cell types, BH3 mimetics should be relevant to diverse cancers, because they engage a universal apoptotic control mechanism.

We conclude that the advent of BH3 mimetic drugs represents a notable advance in cancer treatment. Extending their applications to multiple tumour types, including metastatic solid tumours, and optimising their integration with conventional and targeted therapies should lead to greatly protracted remissions and even curative therapies for a number of cancers.
